# Peripheral Blood Mononuclear CD133 mRNA Levels Correlates with Response to Treatment in Patients with Gastrointestinal Stromal Tumors

**DOI:** 10.1371/journal.pone.0055520

**Published:** 2013-02-07

**Authors:** Yanhong Deng, Manal M. Hassan, Jianwen Mo, Edward H. Lin

**Affiliations:** 1 Seattle Cancer Care Alliance, Fred Hutchinson Cancer Research Center, University of Washington, Seattle, Washington, United States of America; 2 Department of Medical Oncology, The Sixth Affiliated Hospital, Sun Yatsen University, Guangzhou, China; 3 Department of Gastrointestinal Medical Oncology, The University of Texas M. D. Anderson Cancer Center, Houston, Texas, United States of America; Johns Hopkins University, United States of America

## Abstract

**Background:**

CD133 is a marker that identifies/enriches cancer stem cell implicated in tumor initiation. We hypothesize that changes in the CD133 mRNA expression levels and vascular endothelial growth factor (VEGF) may correlate tumor response in GIST.

**Methodology/Principal Findings:**

After informed consent, we obtained peripheral blood samples from 24 evaluable patients with gastrointestinal stromal tumors (GIST). There were 7 -paired samples before and after treatment, We measured CD133 mRNA levels by real time RT-PCR method and vascular endothelial growth factor (VEGF) levels by ELISA. All measurements were done in duplicates in two separate experiments. The treatment resulted in significant reduction of CD133 mRNA expression (p = 0.048) as well as the level of VEGF (p = 0.003). The mean CD133 mRNA levels for GIST patients was 615. We found no correlation between the CD133 mRNA levels and VEGF levels. (p = 0.826). Logistic regression analysis suggested a relationship between elevated CD133 mRNA levels and fitted probability of eventual progressive disease (PD) and mixed response at 37% for CD133 mRNA of 2.25, and the probability of eventual PD/MR is 84% for a CD133 of 2072 (p = 0.08).

**Conclusions/Significance:**

CD133 mRNA expression levels in GIST patients measured by real time RT-PCR assay appeared to correlate with tumor response to surgery or imatinib and may be used to predict tumor progression. Additional prospective studies are warranted.

## Introduction

Gastrointestinal stromal tumors (GISTs) are a paradigm for the development of personalized treatment for cancer patients. The nearly simultaneous discovery of a biomarker that is reflective of their origin and the presence of gain-of-function kinase mutations in these tumors set the stage for more accurate diagnosis and the development of kinase inhibitor therapy. Subsequent studies of genotype and phenotype have led to a molecular classification of GIST and to treatment optimization on the basis of molecular subtype. Further improvements in GIST treatment may require targeting GIST stem cell populations [1].

Cancer stem cell theory proposed that a subset of cancer cell is responsible for the initiation of drug resistance and tumor progression [2] As this theory has recently gained traction in number of epithelial cancers, cancer stem cells have also been identified from tumors of mesenchymal origin [3]. GIST is believed to derive from mesenchymal stem cells of embryonic mesoderm and displays equally vast array of tissue heterogeneity as cancers of epithelial origin, and hematopoietic systems, which are derived from the embryonic ectoderm and endoderm respectively [4].

CD133, is a 120 kDa five-trans-membrane glycoprotein initially found on hematopoietic stem cells,. CD133 can also represent a subset of cancer stem cells in a variety of the epithelial tumors and most recently in sarcoma [5], [6]. Interestingly, elevated expression of the CD133 in the tumor are associated with increasing poor clinical outcomes in a varieties of epithelial cancers [7] as well as in GIST [8] The range of CD133 expressions among different types of cancers varies greatly in a range 1–25% of the primary tumors [9].

We and Mehtra showed that elevated CD133 mRNA levels in the peripheral blood mononuclear cells are associated with the progression and poor survival in colon cancer and other solid tumors [7], [10] To date, no study had been performed on the effects of treatment on circulating CD133 mRNA expression in patients with GIST. We therefore decided to test if peripheral blood CD133 mRNA may be used as surrogate of the markers for treatment response assessment and surveillance.

## Materials and Methods

MD Anderson Cancer Center Institutional Review Board approved the study. All patients had signed the consent form for this laboratory-based study (LAB02-433). Twenty-four patients with confirmed diagnosis of gastrointestinal stromal tumors were recruited. Many of participants were enrolled in a phase III randomized inter-group trial S0033, Clinicaltrial.gov, NCT00009906. Majority of the patients had already received imatinib for almost 2 years prior to the current study. No treatment naive patients were recruited in the study. All study participants were subjects to 15 ml of peripheral blood draw on one or two different occasions. The median duration between two blood draw was 56 days with range from 21 days to 65 days. 7 patients had paired samples measured twice, the average duration between the two times was 2 months.

### CD133 mRNA Detection

We performed two-step Real-Time RT-PCR first performed the RT-PCR to generate cDNA from patients, which can be used in Real-Time PCR to detect CD133 expression levels in samples. RT-PCR was performed in a total volume of 20 µl containing 0.5 µl of oligo(dT) and hexamers, 1 µg RNA, 2 µl 10× Buffer, and 25 mM MgCl_2_, 10 mM dNTPs, 0.1 DTT, 40 U RNaseOUT and DCEP-treated ddH_2_O. The thermal cycle conditions included 10 min at 25°C, 50 min at 45°C and 70°C 15 min, followed by 40 cycles of 95°C for 45 sec and 60°C for 1 min and 72°C for 1 min.

Real-Time PCR was performed in total volume of 50 µl containing 25 µl 1× SyberGreen Mix-buffer, 1 µl Rox Refeence Dye, 6 mM MgCl_2_, 100 mM KCl, 400 µM dATP, dCTP, dGTP and 400 µM dUTP, 40 mM Tris-HCl, 6 mM300 nM each primers, 2 µl cDNA template for each reaction, followed 95°C for 10 min and 45 cycles of 95°C for 45 min, 55°C for 1 min and 72°C 45 sec.

All reagents used for PCR were purchased from Invetrogen, or Stratagene, and using Stratagene MX-3000 Real-time PCR System.

Data interpretation: the amount of target, normalized to an endogenous reference (GAPDH) and relative to the positive control is defined by the Ct method. The formula is applied as follows: Target amount = 2^−Ct^_,_ where Ct = {[Ct(CD133 sample)−Ct(GAPDH sample)]−[Ct(CD133 calibrator)−Ct(GAPDH calibrator)]}. The assays were run on duplicates in two separate experiments and averages of the two experiments were then used in the final analysis.

### VEGF Detection

We used a complete kit (AssayDesigns, Inc.) for the quantitative determination of human VEGF in plasma of patients. We established standard series using a recombinant human VEGF standard. Wash ELISA microtiter plates once with dd-H_2_O, add 200 µl /well PBS:2% BSA, incubate 1 hour at 37°C, wash 6 times with dd-H2O, add 50 µl/well plasma diluted in PBS:1%BSA. Then incubate for 2 hr at 4°C, wash 9 times with dd-H2O, add 50 µl/well anti-human IgG:HRP diluted in PBS:1% BSA, incubate for 28 hr at 4°C, wash 6 times with dd-H_2_O, wash once with carbonate buffer, add 50 µl/well working substrate solution, 0.5 ml 4.0% OPD, 5 µl 30% H_2_O_2_, 1.0 ml 10× Substrate buffer, 8.5 ml dd-H_2_O. Incubate for 30 minutes at room temperature, add 25 µl/well 4.5N Sulfuric Acid. Read data at A450 nm by Packard SpectroCount and calculated to pg/ml for each sample by Packard I-Smart 2. The assays were run on duplicates in two separate experiments and averages of the two experiments were then used in the final analysis.

### Statistical methods

The peripheral blood samples were collected after informed consent and all results were blinded to the investigators until final analysis. Average of two separates experiments (total of 4 assay readouts) was used for the final statistical analyses. Paired t test were used to analyze the pre- and post CD133 mRNA and VEGF level respectively. Pearson correlation was used to detect the relationship of CD133 mRNA and VEGF levels. Given the skewness of CD133 mRNA levels, we used log CD133 levels and build multivariate model to explore the interactions of CD133 mRNA levels with age, progression of disease, and overall survival in patients with metastatic GIST. We constructed stem and leaf plot and explore the duration of timing of the imatinib therapy versus progression or mixed response using logistic regression and Kaplan-Meir survival method.

## Results

Study enrolled 24 evaluable GIST patients in 2003 (Table 1). Their peripheral blood mononuclear CD133 mRNA and plasma VEGF level were assayed via real time RT-PCR assay and sensitive ELISA (Assay Design Kit) respectively.

**Table 1:** Patient, Tumors and Treatment Characteristics (n = 24).

Category	GIST ( n = 24)
Median age (range)	55(38–70 years)
Sex	
Male	8 (33)
Female	17 (67)

**Figure 1:**
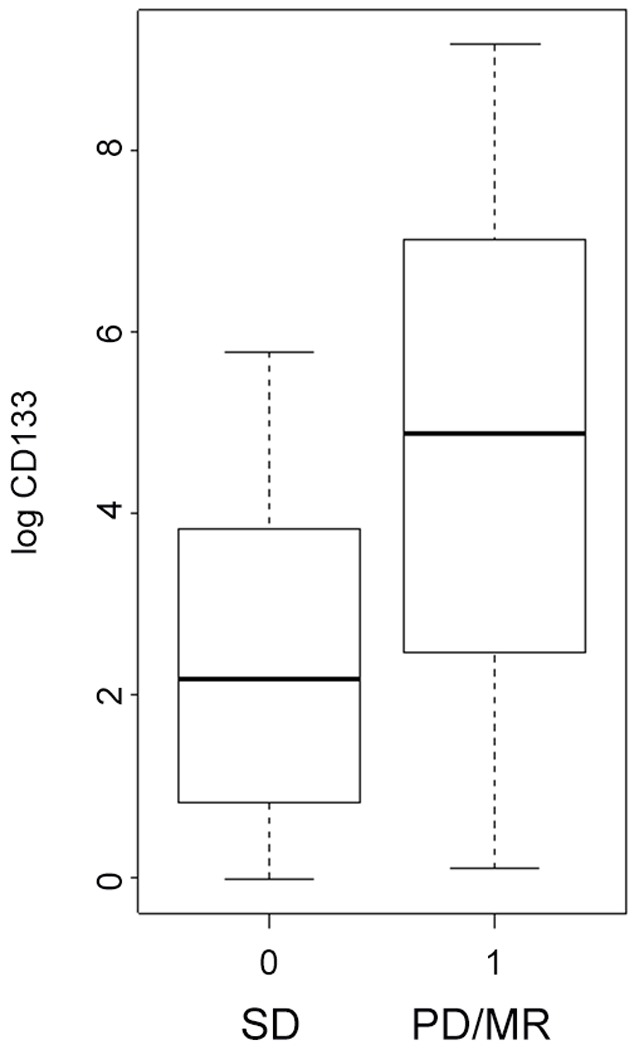
Bivariate relationships of log CD133 mRNA and disease outcomes. Patients with progression disease and mix response had higher CD133 mRNA level than patients with stable disease.

Mean CD133 mRNA levels for GIST patients was 615.17. Mean VEGF levels was 107.42 pg/ml. The results showed that the relative amount of CD133 mRNA expression was significantly lower in the post-treated patients as compared to those same pretreated 7 patients (p = 0.048). The VEGF value was also lower in the plasma of treated patients than that of untreated (p = 0.003). The pre- and post treatment CD133 mRNA and VEGF levels were shown in Table 2. We found no correlation between CD133 mRNA levels and plasma VEGF levels in using Pearson regression analysis, (p = 0.826) (Table 3). We used stem and leaf plot to analyze the duration of imatinib treatment versus progressive disease. We noted a bivariate relationship between CD133 mRNA levels among those patients with stable disease versus those with progressive and mixed responses, Figure 1. The two highest CD133 mRNA levels were found in two patients 3 months (9652) and 15 months (2071) prior to PD (9652). The results highlighted the possibility to use it as a surrogate marker for treatment strategy change in following up the GIST patients who were taking imatinib.

**Table 2:** Mean CD133 mRNA and VEGF levels before and after treatment (n = 7)*.

Mean CD133 mRNA levels		Paired Differences
		*p* value
Before	6.702	*P* = 0.048
After	2.369	
Mean VEGF levels(pg/ml)		
Before	134.117	p = 0.002
After	55.174	

* paired sample test

**Table 3:** Association Analysis Between Two Indicators, CD133 mRNA and VEGF Value*.

Data Items	Two Indicators: CD133 and VEGF	
	Correlation	Sig. (2-tailed)
Average Ratio Value for Both of Untreated to Treated	−0.351	0.183
Average Value for Both of Untreated	−0.028	0.917
Average Value for Both of Treated	0.301	0.258

* Pearson correlation

**Figure 2:**
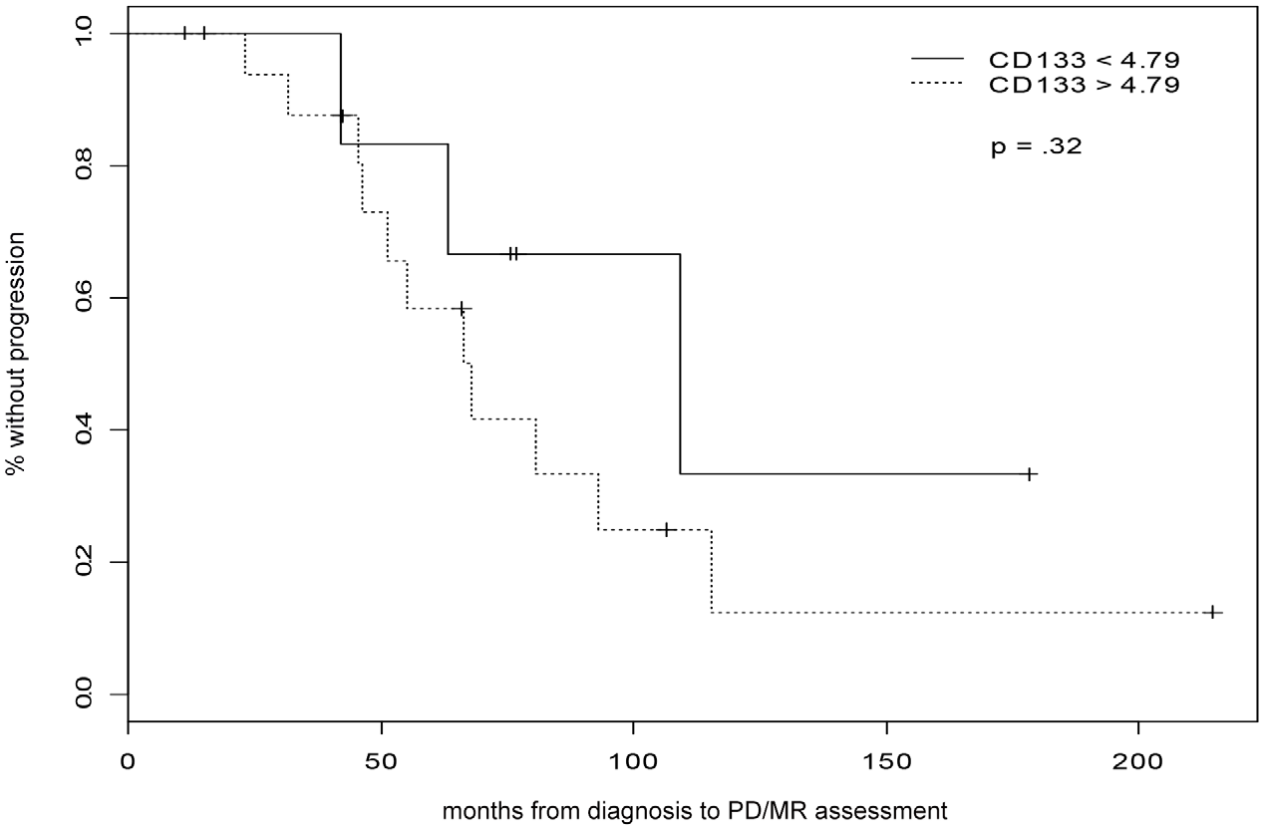
Kaplan Mier progression free survival versus CD133 mRNA cutoff points of 4.79. There was a trend that patients with CD133 mRNA level higher than 4.79 had worse outcome, however the statistical significance was not reached due to the small sample size.

We previously reported that peripheral blood CD133 mRNA at a cutoff point above 4.79 appears to predict colon cancer relapse and poor survival. At the same cutoff point, we found that none of the seven patients whose CD133 mRNA level<4.79 had disease progression except for one patient who had experienced progression 7 months later whereas 14 of 17 (82%) of the patients had CD133 mRNA level>4.7. By a simple logistic regression analysis there may be a relationship between (CD133) and PDMR (p = 0.08), so that the fitted probability of eventual PD/MR is 37% for CD133 of 2.25, and the probability of eventual PD/MR is 84% for a CD133 of 2072. One patient with highest number of CD133 mRNA had died due to very rapid disease progression, which indicated poor prognosis with elevated CD133 mRNA levels. When performed Kaplan Mier analysis on progression free survival using CD133 mRNA levels cutoff point of 4.79, there was a trend that patients with CD133 mRNA level high than 4.79 had worse outcome, however the statistical significance was not reached due to the small sample size, Figure 2 (p  = 0.33).

## Discussion

To our knowledge, this is the first report to demonstrate that peripheral blood mononuclear CD133 mRNA level correlate with the response to imatinib in patients with GIST. Plasma VEGF levels also appear to correlate with reduction in the tumor volume secondary to drug treatment, however, we found no correlation between peripheral blood mononuclear CD133 mRNA levels and plasma VEGF levels. CD133 mRNA when coupled with epithelial markers was able to predict colon cancer relapse and survival [7] The current study highlights the importance of CD133 mRNA as peripheral blood biomarkers for predicting imatinib sensitivity and monitoring the disease progress in the surveillance setting in GIST.

Gastrointestinal stromal tumors (GISTs) have activating KIT or PDGFRA gene mutations. Imatinib mesylate, which targets KIT and PDGFRA, is effective in treating GISTs, but 90% of GIST patients become imatinib-resistant as a result of acquiring secondary KIT mutations. CD133 is a member of prominin family but its functions remains unknown and was found in almost all tissues including retina [11]. Neither tissue nor tumor specific, CD133 identifies with stem/progenitor cells, as well as a host of CSC from variety of tumors notably GBM [12], colon [13], lung [14],and prostate [15] etc. Although, CD133 protein expression was found to be universal expressed in GIST [16], [17], it might not be a good biomarker for CSCs when using protein assay. However, peripheral blood CD133 mRNA level reflected the amount of circulating CD133 positive cells, which often harbors KIT exon 11 mutation representing poor prognosis and chemo-resistant entity [8], might be a good marker for clinical application.

Despite the lack of specific cellular entity of CD133+ cells, their rarity and functional diversity in the peripheral blood argues for the use of RT-PCR assays as primary means of detection which hold certain advantages over flow cytometry or circulating tumor cell technology. Paired samples size and large pre- and post treatment readouts difference in part compensated for the limited sample size in the current study. Low level of CD133 mRNA levels as observed in prolonged imatinib exposed patients suggest a potential treatment effects of imatinib on circulating CD133 mRNA levels. Nonetheless, larger prospective study with serial CD133 mRNA levels performed at diagnosis, pre- and post-treatment and at time of progression along with circulating tumor cells enumerations are needed to assess the value of CD133 mRNA as a surrogate marker.

Lack of correlation of the progression free survival and CD133 mRNA levels may be compounded by a number of factors including lack of clear imaging parameters in 2003 as CHOI criteria was later introduced to address frequent discrepancies of CT scan imaging in interpreting tumor response and progression in patients with GIST tumors treated with imatinib [18]. Again, the sample size is still too small to address these questions definitively.

In summary, peripheral blood mononuclear CD133 mRNA levels represent a potential surrogate for predicting response to imatinib in GIST patients and it is useful in monitoring the disease course during following up. Peripheral blood mononuclear CD133 mRNA levels should be thoroughly studies in conjunction with other biomarkers in prospective clinical trials in GIST.
